# The effect of out-of-pocket costs and financial rewards in a discrete choice experiment: an application to lifestyle programs

**DOI:** 10.1186/1471-2458-14-870

**Published:** 2014-08-23

**Authors:** Johanna O P Wanders, Jorien Veldwijk, G Ardine de Wit, Huberta E Hart, Paul F van Gils, Mattijs S Lambooij

**Affiliations:** National Institute for Public Health and the Environment, Centre for Prevention and Health Services Research, PO Box 1 (Internal postal code: 101), 3720 BA Bilthoven, The Netherlands; Division: Julius Center for Health Sciences and Primary Care, University Medical Center Utrecht, Huispost Str. 6.131, Postbus 85500, 3508 GA Utrecht, The Netherlands; Leidsche Rijn Julius Health Care Centers, Eerste Oosterparklaan 78, 3544 AK Utrecht, The Netherlands

**Keywords:** Out-of-pocket costs, Financial rewards, Lifestyle programs, Discrete choice experiment, Willingness to participate, Diabetes mellitus type 2

## Abstract

**Background:**

Both out-of-pocket costs and financial rewards can be used to influence health related behavior. However, it is unclear which of these two has a larger effect on health related behavior. The aim of this study was to explore the possible difference in effect size between out-of-pocket costs and financial rewards on the willingness of diabetes mellitus type 2 (DM2) patients to participate in a lifestyle program.

**Methods:**

A discrete choice experiment (DCE) questionnaire was sent to 767 DM2 patients in a geographically defined area (De Leidsche Rijn, Utrecht) in The Netherlands and completed by 206 of them. The questionnaire comprised of 18 choice tasks of which 9 contained a financial reward for lifestyle program completion, while the other 9 included out-of-pocket costs for program participation. In a second version of the questionnaire, the order of out-of-pocket cost and financial reward choice tasks was counterbalanced to reduce bias with respect to the position (first or second) of the two types of choice tasks. Panel-mixed-multinomial-logit models were used for data analysis.

**Results:**

Increasing out-of-pocket costs were associated with a decreasing willingness to participate in a lifestyle program and, contrary to our expectations, increasing financial rewards were also associated with a decreasing willingness to participate in a lifestyle program. In addition, this willingness to participate changed to the same extent for both increasing out-of-pocket costs and increasing financial rewards.

**Conclusions:**

As expected, increasing out-of-pocket costs may prevent people from deciding to participate in a lifestyle program. However, offering a financial reward to persuade people to participate in a lifestyle program, may result in decreasing willingness to participate in a lifestyle program as well.

## Background

Out-of-pocket costs and financial rewards are used in an attempt to change people’s behavior, including health related behavior. For instance, out-of-pocket costs like taxes on cigarettes are used to reduce smoking and have been shown to be reasonably successful [1–3]. Examples of financial rewards that are used in an attempt to improve lifestyle include covering membership costs for physical activity programs and rewarding people for performing healthy behavior [4–7]. The use of costs and financial rewards is premised on the notion that humans are rational actors who will consider advantages and disadvantages of the possible options and then choose the option that is best for their (financial) situation. Overall, out-of-pocket costs are thought to have an inhibitory effect on behavior, while financial rewards are thought to stimulate behavior. However, the impact of either out-of-pocket costs or financial rewards on behavior might not be the same. According to Kahneman and Tversky, on average people tend to be aversive to losses [8, 9]. This loss aversion implies that losses and disadvantages have a greater impact on the preferences of individuals than gains and advantages of similar size [9].

A similar response structure may occur when people receive either a financial reward or have to pay at the moment they have to decide whether or not they want to participate in a lifestyle intervention. When accounting for loss aversion, while holding everything else constant, asking potential participants for a financial contribution (out-of-pocket costs) is expected to have a stronger negative effect on their willingness to participate compared to the positive effect of receiving a financial reward of a similar size.

Much research has been conducted on the effect of costs and financial rewards on the outcome of intervention programs (weight loss, physical activity, smoking cessation etc.). These studies show that incentives are effective in general [10–14]. However, participants of these studies are often carefully selected volunteers and they may consequently have a greater motivation to change their behavior than a random selection from the target population. Therefore, we chose not to study the effects of out-of-pocket costs and financial rewards among participants in a lifestyle program, but among a population that is eligible to participate in a lifestyle program. Subsequently, we did not focus on the effect of out-of-pocket costs and financial rewards on the outcome of a lifestyle program, but studied its effect on the preceding step, namely, the willingness to participate in a lifestyle program or not.

The aim of this study is to explore the possible difference in the impact of out-of-pocket costs and financial rewards on the willingness of diabetes mellitus type 2 (DM2) patients to participate in a lifestyle intervention program. This is determined in a discrete choice experiment (DCE) where the stated preferences elicited from scenarios including only financial rewards are compared to the preferences elicited from scenarios including only out-of-pocket costs.

## Methods

### Participants and recruitment

The study population comprised of all DM2 patients of four health care centers with over 20 general practitioners (GPs) in a geographically defined area (De Leidsche Rijn, Utrecht) in The Netherlands. All patients in the study population were primarily treated in primary care. Mentally and/or terminally ill DM2 patients were excluded. In total 767 patients were asked to complete the DCE questionnaire, that was sent along with an accompanying letter from the health care centres. After 3 weeks, a reminder was sent to the patients who had not returned the questionnaire yet. Patients who completed the questionnaire received a voucher of 7.50 euro.

Patient data on ethnicity, gender, age, Hba1C, body mass index (BMI) and use of antidiabetic drugs were retrieved from the Electronic Medical Records by the patient’s general practitioner, and added to the research database anonymously, without further patient identifiers. According to The Dutch National Ethics Board (Central Committee on Research involving Human Subjects) formal testing by a medical ethical committee was not necessary as T2DM patients only had to complete an anonymous questionnaire once, which is in accordance with the guidelines laid down in the Declaration of Helsinki.

### Discrete choice experiment

Discrete Choice Experiments (DCE’s) are used more and more in public health research to estimate the willingness of people to participate in interventions or medical treatments and to provide information about the components of that program that are important for people in their decision whether or not to participate [15, 16]. The DCE methodology is based on the Random Utility Theory and assumes that any intervention or medical treatment can be described by its characteristics (i.e. attributes; such as costs). The individual’s preference for an intervention or treatment is dependent on the levels (e.g., 75 or 100 euro) of those attributes [15, 16]. By varying the levels of the attributes, different scenarios are constructed. Respondents are provided with at least two scenarios (i.e.,choice tasks) simultaneously, they then have to choose the scenario they prefer most. Each respondent is asked to complete a series of such choice tasks. The relative importance of the different attributes and levels can be estimated, providing information on the elements of the intervention that are most important in deciding whether or not to participate in the intervention.

### Attributes and levels

**Figure 1:**
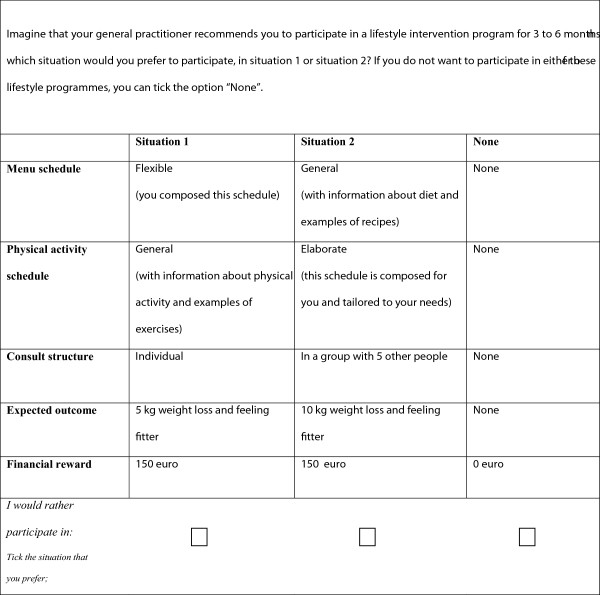
**Example of a choice task.** Figure 1 shows an example of a choice task with a reward for the “money” attribute. Participants choose the situation they most prefer or choose ‘none’ (opt-out) if they do not want to participate in either of the two situations.

A DCE consists of several choice tasks and in this study each choice task comprised of two hypothetical scenarios (unlabelled) and an opt-out option (see Figure 1 for a choice task example). The opt-out option was included because it most closely reflects the situation that patients are in, i.e. voluntary participation in lifestyle programs. Each scenario is described in terms of attributes of the lifestyle program. Each attribute has several levels. To determine the attributes and levels in this study, a stepwise manner was used. First, a literature study was done to compose a list of barriers and facilitators of DM2 patients for participating in a lifestyle intervention [17–27]. Then, the obtained list of barriers and facilitators was discussed using expert interviews (n = 3). Finally, 4 focus groups with DM2 patients (n = 24) were conducted [28] to ensure that the most important attributes for the decision-making process of DM2 patients were included and that the right levels were chosen for each attribute. This resulted in the selection of five attributes for the current DCE: money, menu schedule, physical activity schedule, consult structure and expected outcome. These attributes are the same as the attributes described by Veldwijk et al. [29], since the development of the DCE in this study is based on the same literature study, expert interviews and focus groups with DM2 patients as the DCE described by Veldwijk et al. Each attribute contained three levels, except for the “money” attribute that contained 6 levels (Table 1), either representing financial rewards (75, 150 or 225 euro) for completing the entire program that lasts 3 to 6 months or representing out-of-pocket costs (75, 150 or 225 euro) for participating in the lifestyle program. Veldwijk et al. used only three levels for the “money” attribute, namely three out-of-pocket costs levels. The maximum amount for the “money” attribute level was based on the compulsory level of co-payment within Dutch health insurance, for any type of health services used in 2012. Therefore we assume that the magnitude of the costs and financial rewards was sufficient to influence behavior. The menu schedule and physical activity schedule attributes described how the goals that DM2 patients wanted to achieve concerning a healthier diet and physical activity were developed. The program participants and a lifestyle coach will compose these schedules together using either of the three following options: “flexible”, “general” or “elaborate”. The flexible schedules will be initiated mostly by the participants themselves, because they will set their own goals and develop their own schedule to reach those goals. Within the general schedules, a lifestyle coach will inform the participants about healthy diets and physical activity schedules. Finally, elaborate schedules will be constructed by a lifestyle coach. This coach will develop the diet or physical activity plan tailored to the wishes and needs of the patient. The fourth attribute is the consult structure attribute that describes in what group composition the consults with the lifestyle coach take place. During these consults, participants will develop their menu and physical activity schedule and discuss their progress. The three possible levels of the consult attribute are “individual”, “in a group with 5 other people” and “in a group with 10 other people”. The final attribute is the expected outcome attribute, which describes the outcome that participants expect with respect to weight loss when the program will be completed. The levels of this attribute are: “no weight loss but feeling fitter”, “5 kilograms of weight loss and feeling fitter” and “10 kilograms of weight loss and feeling fitter”.

**Table 1:** **Attributes and levels that were included in the DCE and coding used for data-analysis**

Attribute	Levels	Coding
Menu schedule	*Flexible (ref)*	
	*General*	
	*Elaborate*	
Physical activity schedule	*Flexible (ref)*	
	*General*	
	*Elaborate*	
Consult structure	*Individual (ref)*	
	*In a group with 5 other people* who participate in the lifestyle intervention program	
	*In a group with 10 other people* who participate in the lifestyle intervention program	
Expected outcome	*No* weight loss but feeling fitter	0
	Weight loss of *5 kilograms* and feeling fitter	5
	Weight loss of *10 kilograms* and feeling fitter	10
Money^a^	*Financial reward of 75* euro for 3-6 months	0.75
	*Financial reward of 150* euro for 3-6 months	1.50
	*Financial reward of 225* euro for 3-6 months	2.25
	*Out-of-pocket costs of 75* euro for 3-6 months	-0.75
	*Out-of-pocket costs of 150* euro for 3-6 months	-1.50
	*Out-of-pocket costs of 225* euro for 3-6 months	-2.25
Receiving dummy	Need to pay out-of-pocket costs	0
	Receiving a financial reward	1

^a)^within one choice task both situations had a financial reward or out-of-pocket costs for the “money” attribute.

Before completing the choice tasks, respondents were provided with an extensive explanation of the meaning of all attributes and levels as well as an explanation on how to deal with a choice task, accompanied by an example.

A subgroup of 20 DM2 patients pilot tested a draft version of a comparable DCE as used for our study, before it was finished and disseminated among the entire sample, to test whether the wording used in the questionnaire was correct and whether the target population understood the choice tasks. Most of the pilot tests were postal questionnaires and respondents were asked to indicate which questions they did not understand and give suggestions on how the questionnaire could be improved. In addition, three think aloud pilot tests were conducted to also get information on any difficulties respondents experience while answering the DCE. We also asked whether they noticed a change in the choice tasks half way in the DCE. This change in the pilot study was similar to the change of the financial attribute in our DCE (changing from out-of-pocket costs to financial reward scenarios or vice versa). Based on the pilot results, no changes were made to the questionnaire.

### Experimental design and questionnaire

N-Gene software was used to construct an efficient design for the DCE questionnaire. In this D-optimal design, level balance, utility balance and minimal overlap between attribute levels were optimized. It was assumed that there was no interaction between attributes [30, 31]. The DCE in this study comprised of two blocks of 9 choice tasks. The first block of 9 choice tasks contained hypothetical scenarios with out-of-pocket costs for the “money” atrribute and the second block contained hypothetical scenarios with a financial reward (version 1 of the questionnaire). Participants’ initial reference state or reference state induced by completing the first 9 choice tasks could cause bias. Therefore, the two blocks of 9 choice tasks were reversed in a second version of the questionnaire. The choice tasks with out-of-pocket costs and reward scenarios were clearly separated by a sentence indicating that from this point forward the “money” attribute would change compared to the previous 9 questions. To extra emphasize this change, the previous mentioned sentence was printed in a red color and underlined with a red line.

In addition to the 18 choice tasks, questions about age, gender, income, education, current physical activity levels, and questions about participants’ general opinion about lifestyle programs and their intention to participate in such programs were included. Before answering the choice tasks, participants had to indicate their most preferred level of each attribute. Next to the information obtained by the DCE questionnaire, patient information (age, BMI, sex, use of medication, HbA1c values and ethnicity) from medical records was available. These data were combined for all further analyses.

### Statistical analysis

N-Logit 3.0 was used to construct the panel-mixed-multinomial-logit (Panel-MIXL) models that were used for data analysis. This model takes into account the correlation between the answers given by one participant, which was necessary because each respondent completed 18 choice tasks. By using this model, bias in the attribute estimates, caused by this panel structure in the data, was prevented.

The model in equation 1 describes the utility of a specific lifestyle program based on the attributes that were included in the DCE.
1

β_0_ represents the alternative specific constant, β_1_ – β_8_ are the attribute estimates that indicate the relative importance of each attribute, β_9_ is the estimate of the dummy variable (receiving or paying money) and β_10_ is the estimate for the interaction between money and the dummy variable. Both the constant of the model and the expected outcome attribute were included in the model as a random parameter (assuming a normal distribution). The constant was included as a random parameter, because it was expected that respondent preferences regarding lifestyle intervention programs differ a priori. The outcome attribute was also set as a random parameter, because preference heterogeneity was expected based on the large differences in baseline BMI of participants. The non-linear variables were included in the model using effects coding [32]. By means of a spline function, the part-worth utilities for all attribute levels were estimated to determine whether they should be included as continuous or effects coded parameters.

In order to study whether the impact of hypothetical out-of-pocket costs was larger than the impact of a hypothetical financial reward of the same magnitude, a spline was added to the regression model. This spline enabled to test whether the slopes of out-of-pocket costs and financial rewards differed [33]. The spline consisted of a main effect “money”, a dummy variable “receiving dummy” and an interaction variable “money*receiving dummy”. The value of the dummy variable was 0 in the case of out-of-pocket costs and 1 in the case of financial rewards. As a result, the “knot” was put at 0 euro. This “knot” indicated the possible bend in the regression slope of the association between hypothetical out-of-pocket costs and financial rewards and the willingness of DM2 patients to participate in a lifestyle program. As a consequence of including the spline, the effect of decreasing out-of-pocket costs on the willingness to participate is represented by the estimate of the variable “money” and since we chose to code the dummy variable 1 for receiving a financial reward, the effect of receiving a financial reward on the willingness to participate is represented by the sum of the main effect “money” and the interaction variable “money*receiving dummy”.

## Results

### Study population

In total 206 respondents (response rate 26.9%) completed the questionnaire. Of this sample 53.4% completed version 1 of the questionnaire (out-of-pocket costs first, financial reward thereafter) and 46.6% completed version 2 of the questionnaire (financial reward first and out-of-pocket costs thereafter). All together, 2998 observations (i.e. choice tasks answered) were included in the analysis. Almost half of the respondents stated that a lifestyle program is useful or very useful, but only 24.8% believed that they probably or certainly wanted to participate in a lifestyle program (Table 2).

**Table 2:** **Description of the study population**

	Respondents			Non-respondents		
	*N*	*Mean*	*Percentage*	*N*	*Mean*	*Percentage*
		(SD)			(SD)	
Age (years)	205	61.6		562	61.2	
		(11.5)			(12.6)	
Gender (male)	205		54.1	562		52.7
Ethnicity (West-European origin)	205		64.4	562		56.0
HbA1c (mmol/mol)	194	52.6		499	54.6	
		(10.1)			(12.2)	
Medication (using antidiabetic drug)	205		81.0	562		81.7
BMI (kg/m^2^)	200	29.9		533	30.4	
		(5.4)			(5.5)	
General opinion about lifestyle programs	205					
Very useful			14.5			
Useful			34.0			
Neutral			45.5			
Not so useful			2.0			
Not useful at all			4.0			
Intention to participate in a lifestyle program	200					
Certainly not			21.0			
Probably not			35.1			
I do not know			19.0			
Probably			14.6			
Certainly			10.2			

Of the respondents, 64.4% was of West-European origin and 54.1% were male. The respondents had a mean age of 61.6 (SD 11.5) years and a mean BMI of 29.9 (SD 5.4) kg/m^2^. The mean HbA1c was 52.6 (SD 10.1) mmol/mol and 81.0% used antidiabetic drugs. A non-response analysis (Table 2) showed that the age, BMI, sex and use of diabetes medication were similar between responders and non-responders. HbA1c values were significantly higher (54.6 (SD 12.2) mmol/mol) and the percentage of Western Europeans was significantly lower (56.0%) among non-responders.

### Comparing the effects between a hypothetical financial reward and out-of-pocket costs on the willingness to participate in a lifestyle intervention program

Three attributes “consult structure”, “expected outcome” and “money” were found to influence the willingness of DM2 patients to participate in a lifestyle program (Table 3). Since the focus of this study is on the influence of costs and financial rewards, the remaining part of the results section will only describe the results with respect to the “money” attribute.

**Table 3:** **Attribute estimates (standard errors) of the Panel-MIXL including the spline**

Attribute		Estimates	SE	P-value
Constant		-0.291	0.185	0.115
	SD constant	0.939	0.563	0.095
Menu schedule	Flexible (ref)	-0.027		
	General	-0.034	0.060	0.570
	Elaborate	0.061	0.063	0.331
Physical activity schedule	Flexible (ref)	-0.111		
	General	0.034	0.057	0.546
	Elaborate	0.077	0.052	0.142
Consult structure	Individual (ref)	0.087		
	In groups with 5 other patients	0.158	0.059	0.008
	In groups with 10 other patients	-0.245	0.074	0.001
Expected outcome		0.059	0.013	0.000
	SD expected outcome	0.091	0.047	0.051
Money^a^		0.615	0.122	0.000
Receiving dummy		-0.169	0.244	0.490
Interaction^b^		-1.081	0.147	0.000
Money *Receiving dummy				

^a)^The estimate “money” represents the attribute level estimate of the effect of out-of-pocket costs on the willingness to participate. The less people have to pay for lifestyle intervention program participation, the more willing they are to participate in such a program (Figure 2).

^b)^For the effect of receiving a financial reward on the willingness to participate the estimate of the interaction variable “money*receiving dummy” is added to the main effect “money” (estimate receiving a financial reward=0.615 + (-1.081) = -0.466) (Figure 2).

**Figure 2:**
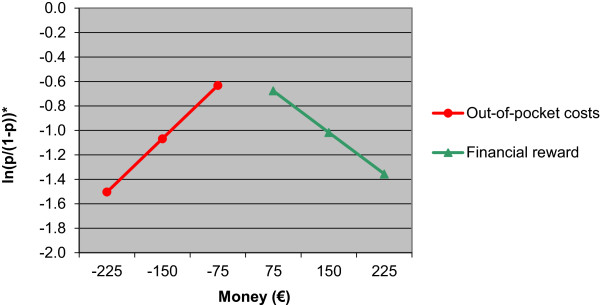
**Influence of costs and financial rewards on the willingness to participate in lifestyle programs.** Figure 2 shows that when out-of-pocket costs for lifestyle program participation increase, the willingness to participate decreases and that when financial rewards for lifestyle program completion increase, the willingness to participate also decreases. *p is the chance that a respondent wants to participate in a lifestyle intervention program.

The attribute level estimate of decreasing out-of-pocket costs is represented by the estimate of the variable “money” in Table 3 (estimate = 0.615). This positive attribute level estimate (due to coding) indicates that the less people have to pay for lifestyle intervention program participation, the more willing they are to participate in such a program (Figure 2). The attribute level estimate of a financial reward is represented by the sum of the attribute level estimate of the variable “money” and the interaction estimate of “money*receiving dummy” (estimate = 0.615 + (-1.081) = -0.466). This negative estimate level indicates that the higher the hypothetical financial reward people expected to receive for program participation, the lower their willingness to participate in a lifestyle program was (Figure 2).

Though the absolute value of the attribute level estimate for receiving a hypothetical financial reward was smaller than the absolute value of the attribute level estimate for paying hypothetical out-of-pocket costs (i.e. indication that people would react stronger to costs than to financial rewards), the difference between these absolute values of the attribute estimate levels was not significant (P > 0.05) (data not shown).

To study whether education, age and ethnicity influenced the association between - costs and financial rewards - and the willingness to participate, these variables were added to the model. Study results did not alter significantly upon inclusion of these variables (data not shown).

To study whether the a priori intention to participate, as expressed in the additional questions, influenced the results, an additional analysis was done, using only the data of the respondents who stated that they would probably or certainly participate in a lifestyle program (24.8%). The results of this analysis were not significantly different from the results described above.

## Discussion

This study aimed to investigate the effects of financial rewards and out-of-pocket costs on the willingness of DM2 patients to participate in a lifestyle program. Results showed that increasing out-of-pocket costs are associated with a decreasing willingness of DM2 patients to participate in a lifestyle intervention program and that, contrary to our expectations, an increasing financial reward was also associated with a decreasing willingness of DM2 patients to participate in a lifestyle intervention program. The negative association between out-of-pocket costs and the willingness to participate confirmed our expectations and is in line with previously conducted research [1, 2, 25, 34]. On the other hand, the negative association between a financial reward and the willingness to participate was not in line with previously set expectations that an increasing financial reward would increase the willingness to participate. However, before completing the choice tasks, participants registered what the minimum amount of compensation for participation in a lifestyle program was that they wanted to receive. Also, it was asked what amount they were willing to pay for participation in a lifestyle program. In both questions, 75 euro was the amount preferred most often (62.3% chose 75 euro as level of compensation, and 72.0% chose 75 euro as level of payment preferred most). So these results are in line with the results of our DCE.

The negative relationship found in the current study may be understood by the reciprocity of the social exchange theory [35]. According to this theory, people feel that the more they are rewarded, the more effort they should put in a relationship or work they are rewarded for. In this case, patients may feel that they should put more effort in the lifestyle intervention program to measure up to reciprocity, when the financial reward they receive gets larger. Instead of motivating people by offering a financial reward, a feeling of obligation may scare people off [36].

Another explanation could be that in most lifestyle intervention research, participants are highly motivated, while our respondents, being a cross-section of DM2 patients within a general practice setting, might be not. In order to create a scenario that may be plausible within the Dutch healthcare system, the maximum financial reward was set at 225 euro. It may be that this amount is sufficient to create a feeling of obligation to comply to a lifestyle program (reciprocity), but is too small to compensate for the efforts that are necessary for a lifestyle change. The actual relation between - costs and financial rewards - and the willingness of people (who have not yet initiated action) to participate in a lifestyle program may be that of a cosine (first a negative slope and then moving up). If this is true, and the financial reward levels included too small amounts of money, we only describe the first part of this relation, resulting in a negative slope. The financial threshold to convince these people to participate in a lifestyle program may be much higher than 225 euro, while people, who are already motivated to participate, do not need such a large incentive. However, it is not realistic to think that people would ever receive a financial reward that is much higher than 225 euro, since this would be not affordable in a healthcare setting.

Finally, it is possible that people feel like they are being explicitly controlled or monitored by a financial reward. People may react negatively to this feeling [37, 38].

In addition, it was expected that out-of-pocket costs would have a stronger impact on the willingness to change behavior than financial rewards. Although out-of-pocket costs tended to have a stronger impact on the willingness to participate than a financial reward, this difference in effect-size was not statistically significant. There might be a significant difference, but that might be too small to detect with this sample size. Since the difference (if existing) is small, one could question if this difference is of practical importance.

### Strengths and limitations

A strength of this study was that the questionnaires contained both reward and out-of-pocket costs scenarios, so both types of choice tasks (with out-of-pocket costs and financial rewards) were completed by the same respondents. Therefore, the comparison between the effects of costs and financial rewards was not biased by patient characteristics or selective response. An additional strength, as already mentioned in the methods section, is that the position of the financial reward and out-of-pocket costs choice tasks was counterbalanced in the second version compared to the first version of the DCE. However, some limitations should be mentioned as well.

First, even after sending a reminding letter, the response rate of 26.9% was relatively low, resulting in a study population of 206 DM2 patients. However such a response rate is comparable with response rates of other DCE questionnaires sent by (e-)mail [29, 39, 40]. In addition, most published DCE's include between the 100 and 300 respondents, so our sample size falls within this range [41]. Nevertheless the relatively low response rate might influence the generalizability of our results.

Due to cooperation with four health care centers, demographic and disease specific characteristics of both respondents and non-respondents were available. Concerning age, BMI, sex and the use of medication respondents and non-respondents were comparable, but the percentage West-European people differed between the groups (64.4% in responders and 56.0% in the non-responders). However, as ethnicity did not significantly influence the association between - costs and financial rewards - and the willingness to participate, we think that the influence of the overrepresentation of West-European responders was limited.

On the other hand, information about other factors like income, social norm, self-efficacy and attitude towards lifestyle programs that could influence the choices of participants, were not available for non-responders. Therefore, the non-response is likely to be selective, in the sense that DM2 patients who are not interested in a lifestyle program were also less likely to participate in this study. It may for instance be that patients who perceive their own lifestyle to be healthy are not interested in lifestyle programs, and therefore did not participate in this study. However, these patients would in real life probably also not participate in an actual lifestyle program, since they think they have a healthy lifestyle, and are therefore of limited interest for this specific study. Though different magnitudes of costs and financial rewards (ranging from -225 to 225 euro) were tested, previous research suggested that several other factors could influence the effectiveness of costs and financial rewards as well. People’s attitude towards a financial reward might for example also be independently influenced by the type of behavior (simple behavior, like a once-only vaccination or complex behavior, like changing lifestyle), the socioeconomic status (SES) of the responding population, frequency of receiving a financial incentive and the moment that the incentive was given (for example at the beginning or after completion of an intervention) [42]. This last factor refers to the present-biased preferences [43]. Education was taken into account in the analysis as a proxy for SES to adjust for possible differences in results due to SES. However, the frequency and the moment that the financial incentive was given or had to be paid was not specified in this study. Since previous research showed that these two factors are also important for the effectiveness of a financial incentive and costs [42], future research should be conducted to explore these effects.

Moreover, traditionally inhabitants of The Netherlands are used to a health insurance without any co-payment for health care. Only since a few years there is a compulsory level of co-payment within the Dutch health insurance system. When the questionnaires were administered, the maximum amount of co-payment was 220 euro for all health care on a yearly basis. So people in The Netherlands are not used to paying high co-payments for health care. As a result, it may have been difficult for them to value this lifestyle program as offered within a health care setting. Therefore, our results may be less generalizable to countries with health insurance systems where people are used to high levels of co-payments for health care.

A limitation of a DCE in general is that there is limited knowledge if hypothetical choices made in a DCE reflect actual choices as made in real life. The external validity of DCE’s is still under-researched [44]. Therefore, research to test for a possible difference between the influence of hypothetical costs and financial rewards on the hypothetical willingness and the participation rates of such lifestyle intervention programmes in real life, is needed.

## Conclusions

This study shows that increasing out-of-pocket costs for participation in a hypothetical lifestyle intervention program are associated with a decreasing willingness of DM2 patients to participate in that program, and that, contrary to what was expected, an increasing financial reward for participation in a hypothetical lifestyle program is also associated with a decreasing willingness to participate. Increasing out-of-pocket costs may prevent people from deciding to participate in a lifestyle program. However, offering a financial reward to persuade people to participate in a lifestyle program may result in a decreasing willingness to participate in a lifestyle program as well.

## Abbreviations

BMI: Body mass index
DM2: Diabetes mellitus type 2
DCE: Discrete choice experiment
GP: General practitioner
Panel-MIXL: Panel-mixed-multinomial-logit models
SES: Socioeconomic status.
